# Side-chain rotamer changes upon ligand binding: common, crucial, correlate with entropy and rearrange hydrogen bonding

**DOI:** 10.1093/bioinformatics/bts395

**Published:** 2012-09-03

**Authors:** Francis Gaudreault, Matthieu Chartier, Rafael Najmanovich

**Affiliations:** Department of Biochemistry, Faculty of Medicine, Université de Sherbrooke, 3001, 12e Avenue Nord. Sherbrooke, Québec, Canada J1H 5N4

## Abstract

**Motivation:** Protein movements form a continuum from large domain rearrangements (including folding and restructuring) to side-chain rotamer changes and small rearrangements. Understanding side-chain flexibility upon binding is important to understand molecular recognition events and predict ligand binding.

**Methods:** In the present work, we developed a well-curated non-redundant dataset of 188 proteins in pairs of structures in the Apo (unbound) and Holo (bound) forms to study the extent and the factors that guide side-chain rotamer changes upon binding.

**Results:** Our analysis shows that side-chain rotamer changes are widespread with only 10% of binding sites displaying no conformational changes. Overall, at most five rotamer changes account for the observed movements in 90% of the cases. Furthermore, rotamer changes are essential in 32% of flexible binding sites. The different amino acids have a 11-fold difference in their probability to undergo changes. Side-chain flexibility represents an intrinsic property of amino acids as it correlates well with configurational entropy differences. Furthermore, on average b-factors and solvent accessible surface areas can discriminate flexible side-chains in the Apo form. Finally, there is a rearrangement of the hydrogen-bonding network upon binding primarily with a loss of H-bonds with water molecules and a gain of H-bonds with protein residues for flexible residues. Interestingly, only 25% of side chains capable of forming H-bonds do so with the ligand upon binding. In terms of drug design, this last result shows that there is a large number of potential interactions that may be exploited to modulate the specificity and sensitivity of inhibitors.

**Contact:** rafael.najmanovich@usherbrooke.ca

## 1 INTRODUCTION

Proteins bind small molecules as substrates, cofactors and allosteric regulators in order to perform essential cellular functions. As a consequence of induced fit (Koshland, 1958), conformational selection (Rubin and Changeux, 1966) or more likely a combination of both (Csermely *et al.*, 2010), the ligand-bound protein may display a wide gamut of structural changes. These changes can range from large movements of entire domains to small side-chain rearrangements in the binding site.

It is widely accepted that flexibility is essential for protein function (Chothia *et al.*, 1983; Lesk and Chothia, 1984). Studying dynamic aspects of protein structure using nuclear magnetic resonance spectroscopy, for example, is still sufficiently costly and time consuming as to prevent its use in the same scale as X-ray crystallography. X-ray crystallography produces a ‘snapshot’ of a protein that offers little information on dynamic aspects of structure. However, it is possible to compare different ‘snapshots’ to infer dynamic properties of the protein. For example, one can compare two structures of the same protein crystalized in different conditions, say in the bound (Holo) and unbound (Apo) forms. Although this type of comparison is neutral with respect to the mechanism of binding (induced-fit or conformational selection), it makes possible, given the amount of available data, to perform statistically significant large-scale studies of conformational changes associated to ligand binding.

Understanding the factors that affect protein flexibility has important practical applications, as efforts in trying to simulate flexibility (even restricted to side-chain movements) have been limited so far by the drastic increase in the size of the associated conformational search space. For example, docking algorithms are an example in which the flexibility of the protein can have a drastic impact on the results. As such, any knowledge that can be applied to decrease in a sensible way the size of the search space is advantageous.

Our earlier study (Najmanovich *et al.*, 2000), among the first statistical studies of side-chain flexibility upon ligand binding, uncovered general features regarding side-chain rearrangements. We used a dataset of paired X-ray structures of the same protein in bound and unbound forms comprising 353 complexes representing 153 different proteins. We used a 60° dihedral angle difference threshold to denote the occurrence of conformational changes. Using this definition, we showed that up to three flexible residues account for the conformational changes observed in 85% of all binding-sites studied and that different amino acid types have different probabilities to be observed in conformationally different states upon binding.

More recently, a study revisited the question of side-chain flexibility upon binding measuring Cartesian coordinate differences (Gutteridge and Thornton, 2005) using an ensemble of 60 complexes. The authors measured root mean square differences (RMSD) of Cartesian coordinates between atoms in the Apo form and those in the Holo form bound to all ligands necessary for the reaction at hand. For example, if an enzyme requires a cofactor or multiple substrates, the Holo form used for comparisons was that containing all such ligands present. The authors calculated the RMSD between: (i) all atoms in the superimposed structures; (ii) atoms belonging to residues that bind the ligands or (iii) those implicated in catalysis. As a control, the authors calculated the RMSD between pairs of different Apo form structures of the same proteins. On the basis of histograms of such RMSD distributions, the authors conclude that conformational changes on substrate binding can be quite subtle (RMSD *<* 1 Å) and that such movements are of the same magnitude as those observed between Apo forms. As recognized by the authors, the RMSD is a problematic quantity when detecting local movements, as the RMSD is a global quantity that measures an average over all residues/atoms considered. For the same reason, RMSD distributions for sets containing largely different numbers of atoms should not be compared (such as binding site and catalytic site residue sets). Interestingly, the authors suggest that catalytic residues are more rigid than binding site (non-catalytic) residues. However, the use of the global RMSD as a measure of flexibility makes it difficult to judge the extent of local movements taking place.

Using an indirect approach, a study explored side-chain movements upon binding using success in flexible docking simulations to evaluate the extent of movements required upon binding to accommodate ligands within 2.5 Å of the observed crystallographic solution (Zavodszky and Kuhn, 2005). On the basis of the docking results obtained using a dataset of 63 complexes representing 20 different proteins, the authors proposed the minimal rotation hypothesis. This hypothesis states that protein side-chains move as little as necessary in order to accommodate ligand binding, i.e. involving mostly modest changes of less than 15°. Two caveats in this study may limit the extent of their conclusions. First, the small number of unique proteins studied. Second, and perhaps more important, the fact that the authors use success in detecting a docked conformation of the ligand (in the presence of side-chain flexibility) within 2.5 Å of the crystallographic solution as a measure of the importance of flexibility upon binding. It is unclear if dihedral angle changes of *<*15° would suffice to accommodate the ligands in their precise experimentally observed positions with less permissive RMSD than the 2.5 Å used. Therefore, while the minimal rotation hypothesis may be a useful approximation in docking simulations if validated in a larger dataset, it still remains to be seen if it is applicable to explain side-chain flexibility upon binding.

Our earlier study (Najmanovich *et al.*, 2000) was based on dihedral angle differences. However, such a measurement is in fact a surrogate measure, a more direct measurement of side-chain conformational changes that is more properly anchored on thermodynamic principles is required to measure the extent with which true side-chain conformational changes are observed as a result of ligand binding. This is particularly important in light of the results of the articles discussed above (Gutteridge and Thornton, 2005; Zavodszky and Kuhn, 2005) suggesting that small-scale changes are sufficient to account for side-chain flexibility in ligand binding.

Side-chain rotamers are defined as particular combination of side-chain dihedral angle ranges. Average dihedral angle combinations of frequently observed conformations represent energetically favorable states. The classification of side chains into rotamers using side-chain rotamer libraries (Dunbrack and Cohen, 1997) makes it possible to determine the extent of side-chain conformational transitions between energetically distinct states as a result of ligand binding. Such libraries also tabulate the probability with which different rotamers are observed. Therefore, in principle rotamer libraries can also help decrease conformational search space with the use of rotamers as representative conformations of energetically favorable states.

The amino acids observed in any particular protein-binding site are the result of natural selection fulfilling a myriad of constraints. These include structural constraints in terms of the geometry of the binding site and particularly of catalytic residues as well as physico-chemical constraints related to binding. The evolutionary selection of binding site amino acids involved in binding is due in part also to the flexibility of side chains as it impacts the specificity with respect to the natural (cognate) ligands as well as the selectivity of the binding site in terms of preventing the binding of competing ligands within the cellular milieu (Najmanovich *et al.*, 2008). Therefore, the conformational changes observed when studying the binding of non-cognate ligands might not necessarily be equivalent to those seen with cognate ligands. Therefore, in order to draw conclusions about the extent of side-chain flexibility upon binding one has to restrict the analysis to proteins bound to cognate or near cognate ligands. As protein–ligand complexes present in the protein databank (Berman *et al.*, 2007, 2000) contain both cognate and non-cognate ligands, it is important to consider this factor when selecting a dataset (Bashton *et al.*, 2007). None of the previous studies mentioned above, with the exception to some extent of the work by Gutteridge and Thornton (2005), takes this factor in consideration.

In order to clarify the extent of side-chain flexibility observed upon ligand binding, it is necessary to perform an analysis using a non-redundant dataset of protein cognate–ligand pairs as large as possible to increase the statistical significance of the results. Such an analysis must also be based on the detection of side-chain rotamer transitions as a way to assure that observed conformational changes involve the transition across energetic barriers.

In this study, we built a curated non-redundant dataset of pairs of X-ray protein structures, representing the cognate–ligand bound (Holo) and unbound (Apo) forms of the same protein, in order to study multiple aspects of side-chain flexibility upon ligand binding. The curation involves the filtering or correction of potential factors (such as the assignment of atoms during refinement) affecting the quality of the structures. The steps involved in creating a non-redundant dataset assure that our conclusions are not biased by over-representation of particular proteins or small samples. The non-redundant dataset created as part of this work could be used as a docking benchmark dataset particularly when the effect of side-chain flexibility needs to be taken in consideration in the analysis of the performance of docking algorithms.

We study many aspects of side-chain flexibility by addressing the following questions: (i) What fraction of binding-sites undergoes change upon binding? (ii) To what extent are these movements critical for binding? (iii) How flexible are the individual amino acid side-chains? and (iv) Is the observed flexibility of side chains characteristic of the binding site environment or reflect intrinsic properties of the amino acids?

## 2 METHODS

### 2.1 Definition of database entries

In this study, we only use X-ray protein structures from the Protein Data Bank (PDB) release of October 2010. Since lower resolution structures (above 3.0 Å resolution) raise the level of uncertainty in the assignment of side-chain conformations, only structures with resolution better or equal to 2.50 Å are used for this study.

An entry in the database consists of a pair of structures of a given protein and a particular ligand that is bound to one of the structures, the Holo form for that ligand, while the second protein structure of the same protein represents the Apo form with respect to that ligand. The sequences of the Holo and Apo forms must be identical (100% sequence identity) over at least 80% of the length of either (overlap). These criteria allow us to detect different structures of the same protein in cases where there are small differences in the N and C termini of the proteins, such as when a particular domain of a multi-domain protein is cloned separately and different groups may choose slightly different domain boundaries or the presence of His-tags or other sequences required for purification.

Our objective in this study is to observe side-chain conformational changes associated to ligand binding. To maximize the chances that the binding ligand is the only factor affecting the observed side-chain conformational changes, in this study we restrict our analysis to pairs of structures which differ by the presence of one ligand and thus are defined as Apo and Holo forms for that ligand. The Apo and Holo forms may be bound to other ligands, as long as such ligands are present in both structures and make the equivalent contacts with the protein. Specifically, we remove cases where such extra ligands have more than 10 different contacts in the Apo and Holo forms.

One problem when working with X-ray structures is that sometimes one or more atoms or entire residues could not be resolved due to uncertainty of atom positioning caused by high movements in the crystal. PDB entries missing any binding site side-chain atoms were removed.

### 2.2 Correction of atom assignments

We filter the dataset to distinguish certain potential artifacts in the assignment of atoms during refinement. The assignment of nitrogen and oxygen atoms in electron-density maps is very challenging due their similarities. In many cases it is not possible except at extremely high resolution or when surrounding atoms help distinguish hydrogen bond donor and acceptor N- or O-containing groups. When the two atoms cannot be distinguished, the outermost dihedral angle may be uncertain by 180° in the cases of Asn and Gln. An analysis of a dataset of highly resolved structures showed that Asn and Gln amides needed to be flipped in 20% of cases (Word *et al.*, 1999). In the context of studying side-chain rearrangements with rotamers that have predefined dihedral angles, erroneous atom assignments may lead to errors in the measurement of the frequency of side-chain rotamer changes. Although high-resolution structures (~1 Å) are unlikely to contain mis-assignments, it would impossible to generate a dataset sufficiently large to derive statistically significant conclusions. In the present work, we ensure atoms are correctly assigned and correct assignments when necessary.

Correcting erroneous atom assignments requires the analysis of hydrogen bonding interactions. We do so by using the program REDUCE that adds hydrogen atoms in standard geometry in order to satisfy physico-chemical constraints (Word *et al.*, 1999). In agreement with the previous study, approximately one-fifth of binding site residues (17% for Asn, 16% for His and 20% for Gln) required a flip in order to avoid the amide clashing with neighboring atoms (Word *et al.*, 1999). Moreover, we remove hydrogen atoms from phosphates and carboxylic groups, as they are more likely to be deprotonated in physiological conditions. In the case of ligands, the HET dictionary provided by the PDB is used to retrieve connectivity information.

### 2.3 Selection of ligands and definition of binding sites

For simplicity, our analysis is restricted to ligands that appear as HETATM in PDB records and otherwise excludes nucleic acids or peptides as the focus of this study lies on the interactions between small molecules and proteins. Furthermore, as we are interested in specific interactions of cognate ligands with binding sites that evolved to bind such molecules, the most common molecules found in crystallization buffers are also excluded from our analysis. Such molecules do not play any role in protein function in the majority of cases and comprise sulphates (SO_4_), phosphates (PO_4_), glycerol (GOL), ammonium (NH_4_), citric acid (CIT), (4s)-2-methyl-2,4-pentanediol (MPD), 2-amino-2-hydroxymethyl-propane-1,3-diol (TRS) and 2-(*N*-morpholino)-ethanesulphonic acid (MES). Although water molecules do play a major role in catalysis, their implication in binding has been studied in detail elsewhere (Barillari *et al.*, 2007; Levy and Onuchic, 2006) and are not considered as ligands in the present work. Finally, covalently bound ligands are discarded, as we are interested exclusively in side-chain movements upon binding as a result of non-bonded interactions.

A few filters are applied to decrease the number of cases involving non-specific binding such as imposing a minimum number of ligand–protein contacts and number atoms in the ligand. The reason for the latter is that very small ligands (five atoms or less) only make a few key interactions with the protein and can bind non-specifically. Moreover, one issue when working with PDB files is that they contain the coordinates of the asymmetric unit, which does not necessarily represent the biological unit of the protein. To circumvent this potential source of errors when using asymmetric units, we only retain ligands that are buried into a protein cavity belonging to a single polypeptidic chain. The fraction of ligand area in contact Fc = SAS_b_/SAS_f_ is used to quantify the degree of burial of a ligand, in which we compare the solvent accessible surface (SAS) of the free ligand (SAS_f_) to the bound ligand form (SAS_b_) in the same ligand conformation. The minimum allowed fraction of ligand area in contact is 0.70.

The binding pocket is defined as the set of residues with at least one atom with surface area in contact with an atom of the ligand. Surfaces in contact between atoms as well as SAS are calculated using a Voronoi Polyhedra analytical algorithm (McConkey *et al.*, 2002) using standard van der Waals radii used for the protein and the ligand atoms as described elsewhere (Tsai *et al.*, 1999). To ensure ligands are specifically bound to the protein, ligands must contain a minimum of five non-hydrogen atoms and to be in contact with at least five residues in the protein.

### 2.4 Generating non-redundant subsets

Using the filters and parameters described in the preceding paragraphs, we obtain a redundant dataset that we call the PRIMARY (PRI) database. Non-redundant subsets are derived from the PRI dataset.

Our objective in developing non-redundant datasets is that every ligand and protein domain combination is represented equally. To do so, ligands are associated to a Pfam domain (Punta *et al.*, 2011) based on the protein domains with which they interact using the PROCOGNATE v.1.6 database (Bashton *et al.*, 2007). In cases where domain assignments for a given complex are not available in PROCOGNATE, a domain is manually assigned by searching the protein sequence using Hidden Markov Models (HMM) against the HMM Pfam library A using HMMer (Finn *et al.*, 2011). Similar to PROCOGNATE, if at least 75% of the total number of contacts of a molecule occur with atoms belonging to a particular Pfam domain, its binding is assigned to that domain. In cases where a molecule has contacts with multiple domains and none has at least 75% of the total contacts, all domains in contact are assigned, leading to multiple entries. The first non-redundant dataset is called PFAM and contains one entry for each different ligand/Pfam domain combination. Different entries could be chosen as a representative for a given ligand/Pfam domain combination. For example, two different human protein kinases bound to ATP and compared to their respective Apo forms could be present in the PRI dataset. In the PFAM dataset, only one representative is retained. The representative Pfam/ligand combination is chosen according to the largest fraction of ligand area in contact (*R*_c_).

One problem with the non-redundant PFAM dataset is that the same or different proteins representing the same Pfam domain and bound to different ligands appear as different entries in the database. To remove this source of redundancy with respect to protein sequences, a more stringent dataset called SEQ was derived from the PFAM dataset. In the SEQ dataset, only entries with protein sequence identity below 50% for a given Pfam domain are retained. As before, the choice between different entries is made based on *R*_c_. Supplementary Figure S1 shows the relationship between the PFAM and SEQ datasets. In this case, one is ultimately loosing some bona fide entries by choosing a representative entry among many containing different ligands.

### 2.5 Side-chain rearrangement analysis

Throughout this study, the Penultimate backbone-independent rotamer library is used as reference for rotamer assignment (Lovell *et al.*, 2000). We use similar filters as the Penultimate backbone-independent rotamer library to filter out low-quality non-rotameric (NR) side-chain conformations. Namely, we eliminate NR side chains with any heavy atom having either an absolute B-factor *>* 40, an occupancy *<*1.0 or containing an alternative conformation.

We use a rotamer-based approach assuming that residues observed in different rotameric states point to conformational states separated by energetic barriers. Residues observed in different rotameric states in the Apo and Holo forms are referred as flexible throughout or rigid otherwise. Rotamers are defined as a combination of dihedral angles. We calculate for each rotatable bond *i*, the dihedral angles (referred as *χ_i_* throughout), using the same definition of atom names and *χ* angles as the one used by Lovell *et al.* (2000). A rotamer of residue type R (*R*_ROT_) from the Penultimate rotamer library is assigned to each binding-site residue (*R*_BPK_), by comparing *R*_BPK_ *χ_i_* to the corresponding *R*_ROT_ *χ_i_*. A rotamer is excluded when *R*_BPK_ *χ_i_* is not within the range *R*_ROT_ *χ_i_*± 30° (unless a specific range is defined for the particular rotamer in the library). After excluding all rotamers that are not applicable, the remaining rotamer is the one to be assigned. In cases where all rotamers are excluded, the side chain is assigned as NR. Cases for which the bound and unbound rotamers are assigned to NR are removed, as it is difficult to judge if the potential side-chain movements represent energetically separate states.

The probability of a residue type *i* to undergo side-chain conformational changes upon ligand binding (*P_i_*) is calculated as previously described (Najmanovich *et al.*, 2000) as follows:
(1)
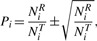

where *N_i_^R^* is the total number of cases in which the rotamers differ and *N_i_^T^* is the total number of residues of type *i* in all binding sites. The second term is the error estimation involved in the measurement.

In some cases, the binding of a ligand can lead to major conformational changes, resulting in significant different protein conformations. In order to simplify our analysis, we choose to limit our study to cases where the average backbone displacement of the binding site is below 2.50 Å (RMSD). Although this threshold may seem a bit permissive, we find that such a threshold offers an acceptable balance as a more stringent threshold leads to a significant loss of data.

### 2.6 Physical constraints analysis

Steric clashes are quantified using a potential (WALL) after superimposition of the Holo andApo structures. This allows us to judge if the Holo ligand pose would be acceptable in the Apo form (referred as Apo-bound throughput) in spite of side-chain rotameric changes. The potential is described as follows:
(2)
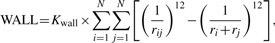

where *K*_wall_ is a penalty constant of value 10^6^ *i* is the *N* th ligand atom; *j* the *M*th protein atom; *r_ij_* the distance between atoms *i* and *j* and *r_i_* and *r_j_* the van der Waals radii. The greater the potential is, the more clashes there are. The potential is similar to that previously described by Sobolev *et al.* (1996), developed to prevent steric clashes in docking simulations. We calculate the differences in WALL term from the Holo to Apo forms. A threshold value is empirically set upon visual inspection of Apo-bound forms to a value of 150 for all ligand–protein contacts, above which the ligand pose in the Apo form is no longer acceptable. This positive value accounts for slight inaccuracies in the radii of ligand atoms. A threshold of 25 is used when considering steric clashes for individual side-chain atoms.

### 2.7 Hydrogen bonding network analysis

Hydrogen bonding is a directional interaction in which an Acceptor ‘A'shares electrons with an hydrogen bound to a Donor ‘D’ (commonly referred as D-H … A). In this study, only strong hydrogen bonding with side chains are considered, i.e. cases where both donor and acceptor are oxygen or nitrogen atoms. Thus, in our study, only the following residues are considered: Arg, Asn, Asp, Gln, Glu, His, Lys, Ser, Trp and Tyr. Considering the prevalence of multifurcation in hydrogen bonding (Sarkhel and Desiraju, 2004), the geometric criteria for their detection are set as *d*_H_*...*_A_ ≤ 2.7 Å (as long as atoms were not clashing: *d*_H_*...*_A_ ≥ 1.5 Å) and *Θ*_D–H_*...*_A_ ≥ 90°. We calculate the number of H-bonds formed between binding site side chains and the following three entities: water, ligand and protein. The difference of H-bonds between the unbound and bound forms is expressed as conserved, gain or loss. Conserved denotes that the total number of H-bonds formed with an entity remains the same after binding whereas a gain denotes new H-bonds are formed after binding. If a residue does not have any H-bond in both forms, it is assigned as conserved. As hydrogen atoms are not explicitly added to water, those acting as donors were treated as if they could always present a hydrogen atom in optimal geometric position.

## 3 RESULTS AND DISCUSSION

In this study, we generated a database that consists of pairs of identical proteins in Holo (bound to a ligand) and Apo (unbound to the ligand) forms to analyze side-chain flexibility upon ligand binding. Our objective is to develop a dataset where, as much as possible, the only factor that influences flexibility is the binding of the ligand in question. The PRI database is the largest dataset that meets our criteria and contains 1812 entries. The database comprises 1270 different crystallographic structures (average resolution of 1.93 Å) defining 163 protein families and 1110 different ligands (Supplementary Table SI). For the purposes statistical analyses, the PRI database is redundant. For example, several proteins were crystallized multiple times under similar experimental conditions. Furthermore, the same PDB might be considered as Holo for a given ligand but as Apo for another ligand.

Two non-redundant subsets (PFAM and SEQ) were generated from PRI. In PFAM, each ligand is uniquely bound to a protein domain (Supplementary Fig. 1), in other words one entry per domain–ligand combination. The same domain can bind different ligands in the PFAM dataset, which can result in different binding site conformations. This subset contains 631 entries defining 884 structures bound to 631 different ligands. The PFAM subset still contains a bias toward particular families of proteins that are over represented in this dataset such as protein kinases (Pfam PF00069) and trypsin (Pfam PF00089) domains, with 143 and 61 cases, respectively, that were crystallized multiple times with distinct ligands.

**Fig. 1.**
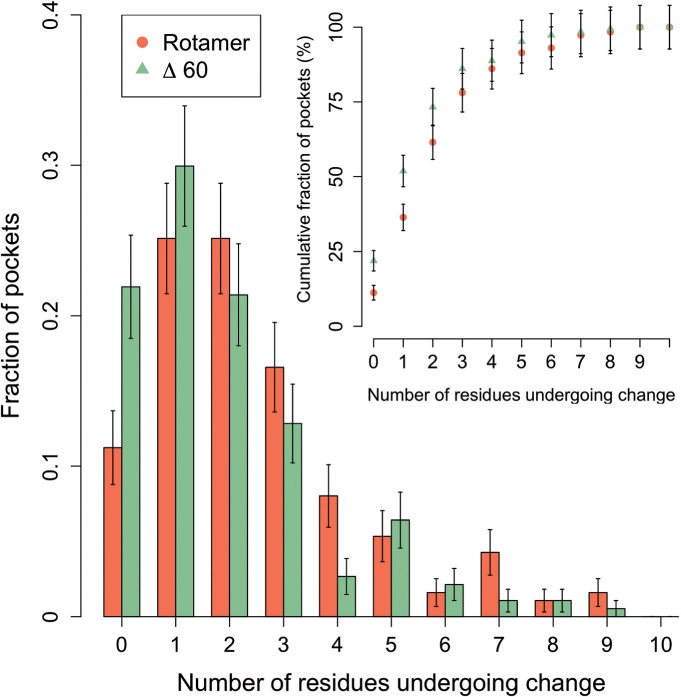
Distribution of the number of residues undergoing conformational changes in binding-sites. Nearly 88% of binding sites contain at least one side chain undergoing rotamer conformational change (orange). At most five side chains undergo conformational change in ~90% of binding-sites (inset). Green bars/triangles show the results obtained using and angular difference threshold of 60° as the criteria for the occurrence of conformational changes

The more restrictive SEQ subset (with 188 entries) removes this bias allowing only one representative for each group of sequences with up to 50% sequence identity belonging to the same Pfam domain. Thus, a protein could appear multiple times in the subset as a result of convergent evolution or if the family diverged sufficiently as to be classified into different Pfam families. For example, the PFAM subset only contains seven protein kinase structures, representing kinases with *<*50% sequence similarity. Statistics for the SEQ subset are shown in Supplementary Table II. Throughout the study, the SEQ subset is used. The list of entries, residues analyzed as well as supplementary data are available at: http://bcb.med.usherbrooke.ca/hap2db.

### 3.1 Side-chain rotamer changes are common

We calculated the fraction of binding pockets undergoing side-chain conformational changes. Residues undergoing changes in binding sites are calculated using a rotamer-based approach as well as the Δ*χ* threshold of 60° used in our previous study (Najmanovich *et al.*, 2000). The Poisson-like distribution obtained (Fig. 1) shows that most binding sites undergo only one rearrangement using the Δ*χ* threshold method and 1 or 2 rearrangements using the rotamer-based method. This result differs from the asymptotically decreasing distribution observed in our previous study (Najmanovich *et al.*, 2000) in which the most common case was that of binding sites with no rearrangements between the bound and unbound forms. There is a significant decrease (2-fold) in the proportion of binding sites where no rearrangements are observed (denoted as rigid henceforth) from what was previously shown. This is particularly interesting, as our methodology in which side chains with very large amplitudes of movements were excluded from the dataset would shift the distribution towards the left-hand side (Supplementary Fig. 2). Therefore, the smaller fraction of rigid binding sites compared to the previous study can be explained by the more stringent constraints used to build the dataset in the current study.

**Fig. 2.**
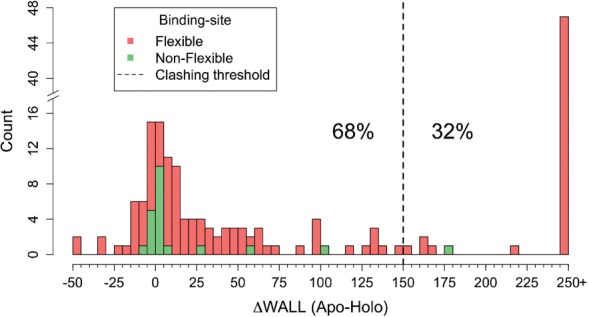
Differences in steric potential (WALL) between Apo-bound and Holo forms. Positive values represent cases where the Apo-bound form is less sterically favorable than the Holo. For clarity purposes, we cap the minimum to −50 and maximum to 250. The empirical threshold value (dashed line) was selected upon visual inspection of all cases in its vicinity

Our previous study looked at each *χ_i_* individually using different threshold dihedral angle values of 45°, 60° and 75° and noticed that side-chain rearrangements are largely insensitive to variation in the threshold. Using the present dataset, we observe a shift in the distributions using higher thresholds (Supplementary Figs 3–4). The use of Δ*χ* thresholds is based on the assumption that large angular differences generally indicate structures belonging to different rotamers. However, the use of *ad hoc* thresholds could overestimate side-chain rearrangements when the Δ*χ* threshold is too low or underestimate the rearrangements when the threshold is too high.

**Fig. 3.**
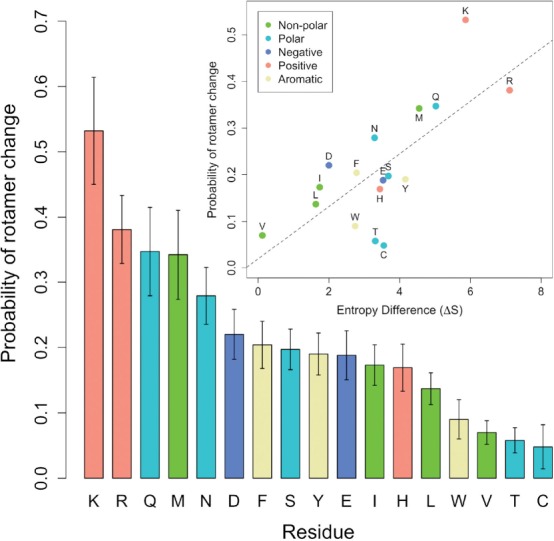
Side-chain flexibility upon binding. The probabilities to undergo rotamer change for different amino acids are shown according to amino acid types. The probability scale correlates with entropy differences (inset)

**Fig. 4.**
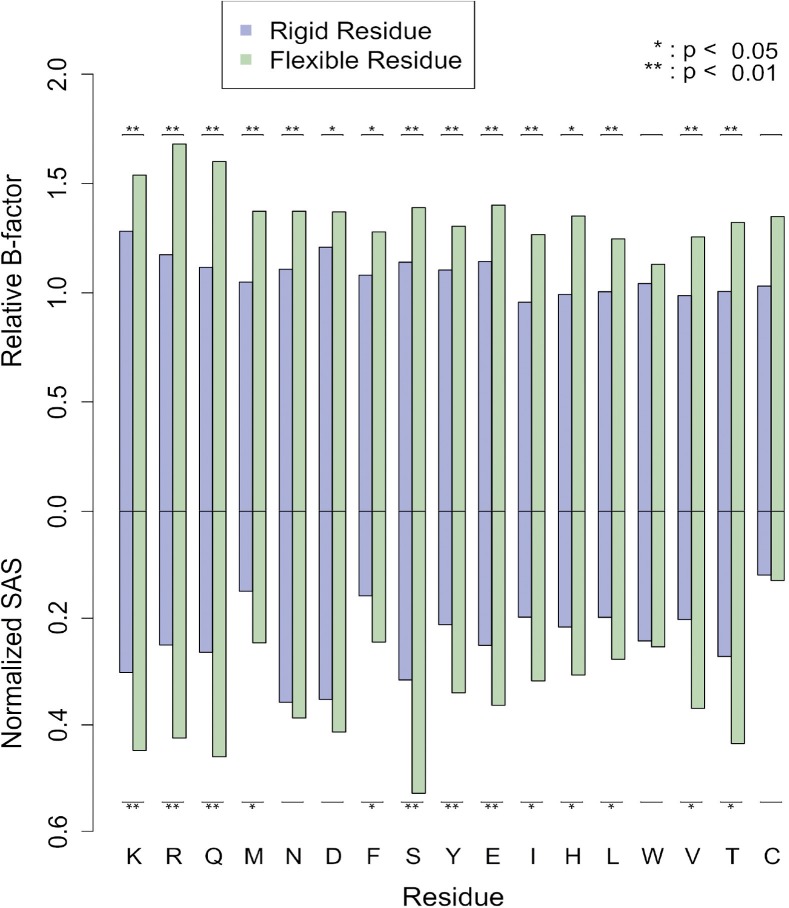
b-Factor and SAS analysis in the unbound form. In upper part, the freedom of movement in the unbound form is quantified using b-factors whereas the lower part represents the normalized solvent accessible surface areas (SAS). Rigid (blue) and flexible (green) residues are compared. Residues are ordered in respect to the flexibility scale. Student *t*-tests are used for statistical significance

The rotamer-based approach used in this study is more appropriate as each residue has its own rotamer classification that renders unnecessary the use of *ad hoc* error-prone thresholds. We account in part for the effect of intrinsic disorder in flexibility when using rotamers as the definition of rotamers consider variations in dihedral angle values.

Overall, our results show that ~12% of binding sites do not change conformation upon ligand binding. In other words, in ~88% of the cases, at least one residue undergo changes which shows the large amount of flexibility that occurs upon ligand binding. Thus, our results show that a significant amount of flexibility occurs upon ligand binding when considering rotamer changes as opposed to RMSD values (Gutteridge and Thornton, 2005). Furthermore, such widespread rotamer transitions represent changes between energetically separate states, thus placing doubt over the validity of the minimal rotation hypothesis outside the context of docking simulations and the small dataset upon which it was developed (Zavodszky and Kuhn, 2005). Although it would be desirable to set all residues as flexible when predicting the structure of protein, it is impractical from a computational point of view considering the exponential increase in size of the search space. However, considering at most five side chains as flexible account for all side-chain rotamer changes in ~90% of binding sites (inset Fig. 1). Therefore, the introduction of side-chain flexibility on a limited number of binding site residues can be seen as a realistic simplification.

### 3.2 Side-chain flexibility is crucial for binding

#### 3.2.1 Steric constraints

We investigated the role of side-chain flexibility from a steric point-of-view using the WALL potential Equation (2) in order to understand to what extent side-chain conformational changes are essential to accommodate the ligand in the binding site. To do so we superimpose the Apo and Holo forms and transplant the ligand to the Apo form, what we call Apo-bound form. We calculate the WALL difference between Apo-bound and Holo forms for all binding sites in the SEQ database (Fig. 2).

As expected, in the subset of binding sites in which no rotamer changes were observed, the Apo-bound and Holo forms show no significant differences in their steric potential for rigid binding sites. Overall, in 32% of flexible cases (28% of all binding sites), side-chain rearrangements are required to accommodate the ligand and to avoid steric clashes. In the context of molecular docking, these results demonstrate the importance of considering side-chain flexibility and define a natural threshold for the accuracy of rigid docking algorithms tested on an unbiased non-redundant dataset.

#### 3.2.2 The minimal rotation hypothesis

The rigid binding site in Figure 2 in which there are severe clashes (the rightmost case in green) is that of Lectin bound to *N*-acetyl-glucosamine (Supplementary Fig. 5). In this case, the residue that contributes the most to the WALL term is at 2.0 Å in the Apo-bound form but and moves just enough (0.7 Å) to avoid clashing with ligand atoms in the Holo structure. However, this small change is brought about by a small rotation that does not represent a rotamer change. When looking at the residue responsible for the most severe clashes in the Apo-bound form (Supplementary Fig. 6), we observe 170 residues with critical steric clashes. Of these, 54 undergo a rotamer change (out of 472 that undergo rotamer change) and 116 have changes insufficient to classify as a different rotamer (out of 1923 that remain rigid). Overall, only in 37 critical cases (2 flexible and 35 rigid side chains) a dihedral angle change of less than 15° is responsible for the change, in agreement with the minimal rotation hypothesis (Zavodszky and Kuhn, 2005) distributed among 34 binding-sites (out of 188). Therefore, the minimal rotation hypothesis is valid in 21.7% of residues critical for binding (whereas ours is in 31.8%) corresponding to 18% of binding-sites. The previous results illustrate the difficulty of introducing side-chain flexibility and that no single approach can describe all observed changes.

**Fig. 5.**
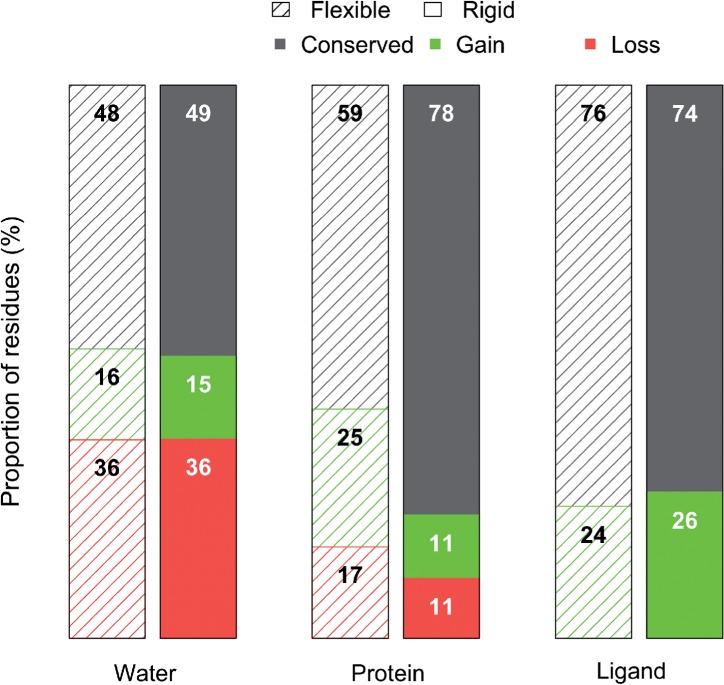
Rearrangement of the Hydrogen bonding network. We plot the proportion of residues that conserve the number, gain or loose H-bonds with water molecules, protein or ligand atoms. We observe a 36% decrease in the number of H-bonds between binding-site residues and water molecules with a concomitant increase of the number of H-bonds for with protein atoms for flexible residues (center left), likely formed with rigid residues as these conserve the number of H-bonds in 80% (center right) of cases. Interestingly, ~75% of residues that could form H-bonds with the ligand upon binding do not fulfill these interactions (right-hand bars)

### 3.3 Flexibility is correlated with entropy differences

In this section, we discuss particular aspects of side-chain flexibility for the different side chains. A total of 2395 residues were considered for the statistical analysis with an average of 141 per residue. Cys has the lowest number of observations with 42 and Leu the highest with 234. Gly and Ala are not considered since they do not have any dihedral bonds as well as Pro, because movement of its side-chain inevitably causes backbone movements. Cys residues participating in disulfide bonds are also excluded as their conformations are constrained. A detailed list of the residues is accessible at our website.

We show in Figure 3 the probability with which each residue undergoes changes (probabilities are listed in Supplementary Table III). In general, the probability of a residue to undergo rotamer change upon binding increases with the number of flexible bonds (in parenthesis) in the following order: Lys(4) *>* Arg(4), Gln(3), Met(3) *>* Asn(2) *>* Asp(2), Phe(2), Ser(1), Tyr(2), Glu(3) *>* Ile(2), His(2), Leu(2) *>* Trp(2) *>* Val(1), Thr(1), Cys(1). There is a 11-fold difference between the probabilities of Cys and Lys. The flexibility scale correlates with that in our previous study (Najmanovich *et al.*, 2000) despite the methodological differences between the two studies and the differences in the procedure used to create a non-redundant dataset.

Lee *et al.* (1994) estimated changes of side-chain conformational entropy (ΔS) in folding/binding for the different amino acids placed at the center of a nine residue *α*-helix. The probability profiles they calculated agree with the frequencies of occurrence of the different side-chain rotamers. The ΔS of Lee *et al.* (1994) correlates well with our flexibility scale (*R*-value is 0.72) apart from a few disparities (Fig. 3, inset). The correlation observed strengthens the validity of the flexibility scale. It also suggests that the closer distance between side chains within binding sites (due to its concavity) does not have a major effect on the entropy of amino acids (as ΔS values were calculated with residues in the surface of an exposed *α*-helix). Therefore, binding-site residues and residues exposed in the protein surface should behave similarly with respect to flexibility. The disparities observed in the correlation could be explained by the contribution of other factors such as local interactions or those with the ligand. The result suggests that flexibility is an intrinsic property of amino acids.

As flexibility correlates with entropy differences, it is interesting to see if there are differences between flexible and rigid residues with respect to quantities related to geometric constraints. In particular, b-factors and solvent accessible surface (SAS) areas. We observe greater SAS and b-factors for flexible residues (Fig. 4). These differences suggest that it should be possible to identify flexible residues in the Apo form.

It was previously suggested that ligand binding induces NR conformations for some amino acid types (Heringa and Argos, 1999). In our dataset, we do not detect any pronounced differences in the number residues in NR conformations between Holo and Apo forms with 127 versus 104 cases (out of 2395 residues). Finally, it was observed that side chains with unfavorable conformations are more mobile than rotameric ones (Carugo and Argos, 1997). Assuming that all side chains observed in NR conformation in the Apo form underwent considerable movements upon binding, we can place an upper bound on the number of residues in NR conformations undergoing large rotations and compare to the number of residues in rotameric states undergoing equally large rotations. We observe 375 NR conformations in the Apo form (an overestimation considering the assumption of movement between NR conformations) compared to 368 rotameric conformations that undergo rotamer change (to another rotamer or an NR conformation). Although we do not make a distinction according to amino acid types, we do not observe that NR conformations are more mobile than rotameric ones.

#### 3.3.1 Hydrogen bonding network

We are also interested to understand how enthalpic factors may affect flexibility. We focus on hydrogen bonds (H-bonds) as this is one of the most important ligand–protein-specific interactions. For simplicity, we focus only on the number of H-bonds conserved, gained or lost upon binding and not on a detailed case-specific analysis of rearrangements of the H-bond network that occurs upon binding. This is because we do not have any information on the pathway (within the H-bond network) or dynamic aspects of such changes for each enzyme as we only use X-ray structures representing the initial and final states of the binding process. Therefore, any cases where the rearrangement of the H-bond network does not change the total number of H-bonds that occur involving protein and water atoms upon introduction of the ligand cannot be detected.

In order to understand the contribution of H-bonding flexibility, we calculated the average number of H-bonds formed with the protein and water molecules. We notice that for His, Arg, Lys, Ser and Thr, they participate in more H-bonds when they are rigid (Supplementary Fig. S7). For the remaining residues, we do not observe a statistically significant difference between flexible and rigid. This shows that upon binding of a ligand, the H-bonding interactions are easily broken. Glu has a lower probability of moving than expected considering its number of flexible bonds. This may be due to the fact that acidic residues have a high tendency to participate in H-bonding (Supplementary Fig. S7).

Although there are small differences on the pattern of H-bonds conserved, gained or lost with water molecules for different amino acid types (Supplementary Fig. 8A and B), the overall rates are essentially indistinguishable between flexible and rigid side-chains (left bars, Fig. 5). Approximately 35% of residues lost H-bonds with water, as expected considering that water molecules within the cleft must be displaced during binding, while others may be necessary for enzyme reaction (Okada *et al.*, 2002; Stillman *et al.*, 1993). Interestingly, only around 15% of residues gain H-bonds with water.

We also compare the number of H-bonds formed within the protein (center bars Fig. 5 and Supplementary Fig. 8C and D). Rigid residues maintain the number H-bonds in 80% of cases compared to 60% for flexible residues. This result supports our rotamer-based approach in the sense that residues that did not significantly move (rigid residues) are less prone to create or lose H-bonds than flexible residues. On the other hand, flexible residues have a slightly higher H-bond gain than loss. This result raises the possibility that flexible residues are replacing H-bonds with rigid residues that occurred before binding with water molecules. We also analyzed the number of H-bonds between the protein and the ligand for flexible and rigid residues. We notice a slight increase in the formation of H-bonds with the ligand for flexible residues (28% over 22% for rigid) for residues that initially formed H-bonds with the protein (Supplementary Table SIV). Interestingly, three quarters of the residues that participate (or can participate) in H-bonds do not create H-bonds with the ligand (right bars Fig. 5 and Supplementary Fig. 8E and F). As such, there is a vast untapped well of potential interactions occurring unfulfilled or fulfilled with protein atoms and/or water molecules that could be exploited in terms of creating new interactions with, for example, an inhibitor.

## 4 CONCLUSIONS

In this study, we show side-chain rotamer changes upon binding are widespread occurring in nearly 90% of binding sites studied. Moreover, in 32% of flexible cases (28% overall), steric clashes would prevent ligand binding in the absence of movements. In the context of molecular docking, one may not succeed in finding the right solution in a large fraction of cases if the protein is not allowed to be flexible. At the same time, it is feasible to introduce side-chain flexibility on a limited number of residues as our results show that five flexible side chains account for 90% of the observed cases. Overall, we studied 2395 side chains. Of these, 472 undergo side-chain rotamer changes or ~20% of all residues. We detected 170 residues whose movements are critical for binding. Among these, 37 undergo dihedral angle rotations of 15° or less distributed among 34 binding sites (18% of all binding sites). Zavodsky and Kuhn (2005) state that ‘Most side chains do not shift to a new rotamer, and small motions are both necessary and sufficient to predict the correct binding orientation and most protein–ligand interactions for the 20 proteins analyzed’. In our dataset, the minimal rotation hypothesis applies to ~22% of (37/170) of all sterically critical residues, while the other 78% are accommodated by larger rotations that may or may not lead to a rotamer change, almost four times the cases covered by the minimal rotation hypothesis.

We observe that side-chain flexibility reflects an intrinsic property of amino acid side chains as it is correlated to configurational entropy differences. Indeed, side chains in the Apo form with higher mobility (b-factors) and exposed surface to the solvent are more likely to undergo side-chain rotamer changes.

Interestingly, we observe a rearrangement of the H-bond network that leads us to propose that upon binding, there is a disruption of H-bonds with water concomitant with a gain of H-bonds for flexible residues, leading to conservation of the number of H-bonds of rigid residues. Furthermore, 75% of residues with potential to form H-bonds do not change the number of H-bonds in which they participate. This points to the possibility that there is a vast potential to develop inhibitors that could take advantage of this undisturbed H-bond network to modulate selectivity and specificity.
